# Neuroprotective Effects of Physical Activity via the Adaptation of Astrocytes

**DOI:** 10.3390/cells10061542

**Published:** 2021-06-18

**Authors:** Grazia Maugeri, Velia D’Agata, Benedetta Magrì, Federico Roggio, Alessandro Castorina, Silvia Ravalli, Michelino Di Rosa, Giuseppe Musumeci

**Affiliations:** 1 Department of Biomedical and Biotechnological Sciences, Human, Histology and Movement Science Section, University of Catania, Via S. Sofia n°87, 95100 Catania, Italy; graziamaugeri@unict.it (G.M.); vdagata@unict.it (V.D.); benedetta89@hotmail.it (B.M.); federicoroggio@gmail.com (F.R.); silviaravalli@gmail.com (S.R.); mdirosa@unict.it (M.D.R.); 2 Laboratory of Cellular and Molecular Neuroscience (LCMN), School of Life Science, Faculty of Science, University of Technology Sydney, Broadway, NSW 2007, Australia; alessandro.castorina@uts.edu.au; 3 Laboratory of Neural Structure and Function (LNSF), School of Medical Sciences, (Anatomy and Histology), Faculty of Medicine and Health, University of Sydney, Sydney, NSW 2006, Australia; 4 Research Center on Motor Activities (CRAM), University of Catania, Via S. Sofia n°97, 95100 Catania, Italy; 5 Department of Biology, Sbarro Institute for Cancer Research and Molecular Medicine, College of Science and Technology, Temple University, Philadelphia, PA 19122, USA

**Keywords:** exercise, astrocytes, neuron, brain functions

## Abstract

The multifold benefits of regular physical exercise have been largely demonstrated in human and animal models. Several studies have reported the beneficial effects of physical activity, both in peripheral tissues and in the central nervous system (CNS). Regular exercise improves cognition, brain plasticity, neurogenesis and reduces the symptoms of neurodegenerative diseases, making timeless the principle of “mens sana in corpore sano” (i.e., a healthy mind in a healthy body). Physical exercise promotes morphological and functional changes in the brain, acting not only in neurons but also in astrocytes, which represent the most numerous glial cells in the brain. The multiple effects of exercise on astrocytes comprise the increased number of new astrocytes, the maintenance of basal levels of catecholamine, the increase in glutamate uptake, the major release of trophic factors and better astrocytic coverage of cerebral blood vessels. The purpose of this review is to highlight the effects of exercise on brain function, emphasize the role of astrocytes in the healthy CNS, and provide an update for a better understanding of the effects of physical exercise in the modulation of astrocyte function.

## 1. Introduction

In the last decades, there has been a growing body of evidence suggesting a positive correlation between a physically active lifestyle and health status. The literature has demonstrated the beneficial role exerted by physical activity to ameliorate general health, by reducing the risk of the most prevalent age-related diseases, including cardiovascular disease, stroke, and diabetes [1,2]. Exercise has also been found to counteract metabolic disorder, inflammation, muscle atrophy, bone and cartilage loss or degeneration, the reduction of aerobic capacity and the progression of several neurodegenerative diseases [3,4,5,6]. Moreover, it is acknowledged that regular and balanced exercising ameliorates antibacterial and antiviral immune surveillance and delays immunological aging. Consistently, very recent studies have underlined the importance of physical activity as an intervention to prevent the progression towards severe forms of COVID-19, based on the fact that exercise can dampen excessive immune responses, thereby eliciting anti-inflammatory functions that promote psychological health [7,8,9]. Exercise represents an effective and successful weapon for optimal aging. Regular physical exercise in healthy adults enhances behavioral performance, memory, attention, processing speed, and executive functions [10]. Moreover, it positively acts on brain function and productivity and decreases the risk of age-related cognitive disorders [11]. Physical exercise promotes neurogenesis and increases the number of neurons, synapses, and pre-and postsynaptic function. It may also increase the size of astrocytes, astrocytic transporter levels and reduce astrocytic degeneration [12]. In the present review, we discuss the impact of physical exercise on brain function, by focusing on the effects of exercise on astrocytes, whose changes might be a key mechanism accounting for exercise-related improvement of cognitive and executive brain functions.

## 2. Beneficial Effects of Physical Exercise on Brain Function

**Figure 1:**
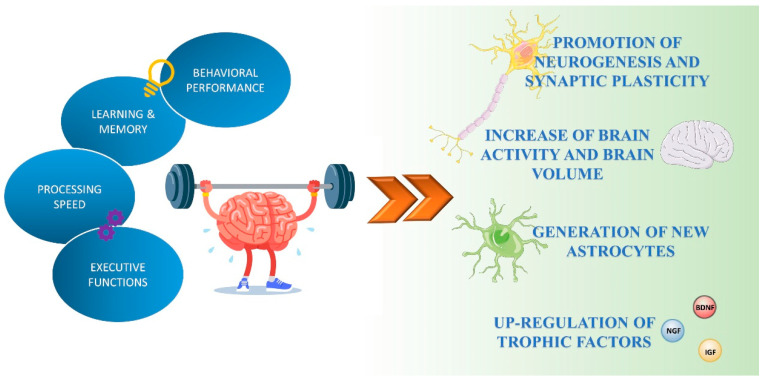
Exercise-mediated effects on brain functions. Exercise enhances cognitive performance by promoting neuronal plasticity, neurogenesis, and astrogenesis, and the production and release of neurotrophic factors.

Exercise has positive effects in nearly all human organs and physiological systems (e.g., metabolic, musculoskeletal, cardiovascular, respiratory, immune, and digestive) [13]. Physical activity also improves brain health and can play an important preventive role in cognitive decline related to aging (Figure 1) [14].

Several human and animal studies have shown exercise-induced structural and functional changes in the brain. Aerobic exercise performed for several months to a year has been shown to increase brain activity and the volume of different brain regions, such as the prefrontal and temporal cortex [15] and the hippocampus [16], suggesting that physical activity can promote “brain rejuvenation” in areas that are critical for cognitive and executive efficiency. Exercise induces neurogenesis and enhances synaptic plasticity [17,18,19]. Over the years, both voluntary and forced exercise protocols have been used to explore the effect of exercise on brain function. Some of this research has been focused on addressing the role of exercise in the hippocampus, a brain area important for learning and memory. In rodent models, voluntary running enhances neurogenesis in the hippocampal dentate gyrus (DG), increases neuronal spine density, and modulates long-term potentiation (LTP), a synaptic strengthening process associated with learning and memory [20,21,22]. O’Callagan and colleagues observed that 7 days of forced treadmill-running exercise induced an increase of LTP in the DG compared to the sedentary controls [23]. Moreover, regular aerobic activity activated hippocampal neurogenesis not only in young mice but also in aged mice [14], suggesting that exercise might be a way of delaying and treating aging-related memory decline.

The neuroplasticity promoted by exercise is at least in part due to the increased expression and release of neurotrophic and growth factors, including brain-derived neurotrophic factor (BDNF), insulin-like growth factor-I (IGF-I), and nerve growth factor (NGF), which are currently considered key proteins during plasticity events that follow exercise [24,25,26]. BDNF is the most widely distributed neurotrophin in the adult mammalian brain. It plays a crucial role in the differentiation of neural stem cells into neurons, influences neuronal plasticity by facilitating the release of neurotransmitters, and shows a neuroprotective effect under adverse conditions, such as excessive glutamatergic stimulation, cerebral ischemia, and neurotoxicity [27,28,29,30]. BDNF, through the activation of its receptor tropomyosin receptor kinase B(trkB), triggers a number of growth and survival-promoting intracellular signaling pathways, including the Ras/MAP kinase, and phosphatidylinositol-3 kinase (PI3K)/Akt cascade, involved in neural plasticity, stress resistance, and neuronal cell survival. Additionally, BDNF prevents neuronal apoptosis by inducing the expression of the antiapoptotic Bcl-2 family members and by inhibiting proapoptotic proteins such as Bax and Bad [31]. Several studies have found that physical activity increases the expression of BDNF and other neuronal plasticity-related genes in the striatum and cortex of young and aged rats [32,33,34]. Furthermore, it has been shown that after three consecutive days of aerobic exercise, the levels of BDNF increase in the rat hippocampus and remain elevated after exercise ends [35]. In humans, serum BDNF levels were found to be increased, along with improvements of certain cognitive functions, after acute cycling exercise [36,37]. Vaynman et al., also found that voluntary exercise not only increased mRNA levels of BDNF but also those of its primary receptor TrkB and the downstream transcription factor c-AMP response element-binding (CREB). Similarly, treadmill exercises significantly increased levels of BDNF, TrkB, and synaptotagmin, a synaptic protein involved in learning and memory function [38].

IGF-I is a neuroprotective agent which plays an important role in brain development, neural survival, and supporting the vasculature [39]. In fact, reduced IGF1 signaling in astrocytes impairs their ability to support neurons under conditions of stress, and this is associated with defects in the mitochondrial respiratory chain of astrocytes [40]. Animal studies have highlighted that running induces the uptake IGF-I by specific groups of neurons [24,41]. Evidence demonstrates that the IGF-I signaling pathway mediates exercise-induced angiogenesis, and stimulates the release of BDNF to promote neurogenesis [24,41,42,43]. Exercise also stimulates the release of NGF, which is normally produced in the hippocampus throughout life and retrogradely trafficked to septal cholinergic neurons. By doing so, it provides a potential mechanism for modulating cholinergic inputs and, thereby, ensuring hippocampal plasticity [44,45]. NGF, through the activation of the PI3K/Akt pathway, plays an important role in promoting neuronal survival and brain plasticity [46,47]. In rodents, moderate-intensity treadmill exercise has been associated with the upregulation of NGF mRNA in the hippocampus and in the cortex [26]. In addition, exercise-induced NGF suppressed apoptotic events in the hippocampus of aging rodents [48,49].

Besides the effects on neurons, and on neurotrophic release, physical exercise is also known to have several effects on glial cells. Among them, astrocytes, representing the major glial cells in the brain, are indispensable to sustain neuronal function. Therefore, the beneficial effects of exercise in the brain should also be seen in the context of astrocytic modulation.

## 3. The Functions of Astrocytes in the Healthy CNS

**Figure 2:**
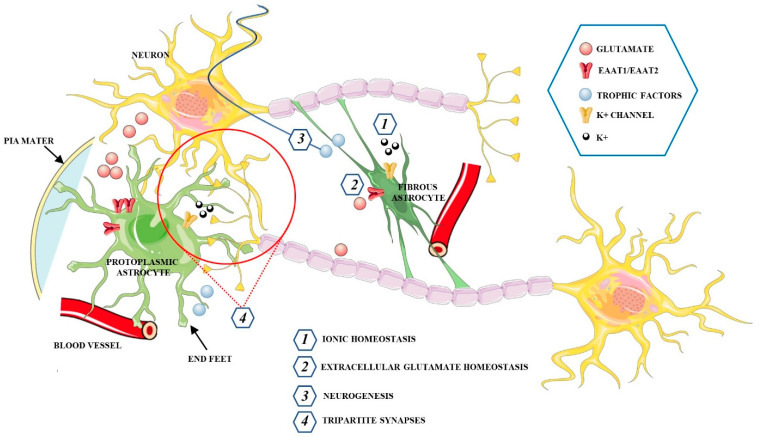
Schematic representation of the main functions exerted by protoplasmic and fibrous astrocytes in the CNS.

Astrocytes represent the most abundant glial cells in the human brain. Although they were represented as a homogeneous population of cells, several data now indicate that astrocytes are highly heterogeneous cell populations [50]. Based on their morphology and spatial organization, astrocytes are classified into two basic subtypes: protoplasmic and fibrous astrocytes (Figure 2) [51].

Protoplasmic astrocytes are widely distributed in the gray matter and show several highly branched and bushy processes, which extend their end-feet to blood vessels and enwrap them to form the glial limiting membrane, which is the outermost wall of the blood−brain barrier (BBB). Fibrous astrocytes, mainly located within the white matter, have a stellate shape with smooth and long processes. This type of astrocyte expresses high levels of glial fibrillary acidic protein (GFAP) as compared with protoplasmic astrocytes, in which the GFAP protein is sometimes found only in the end-feet making contact with blood vessels [52]. Astrocytes associated with neurons, perivascular astrocytes, microglia, pericytes, endothelial cells (EC), and the basement membrane (BM) form the neurovascular unit (NVU), a structure involving multicellular relationships to establish a functional coupling between the brain and blood vessels. Astrocytes localized between neurons and endothelial cells, extend their “end-feet” processes from the cell bodies to surround the arterioles and capillaries, providing almost complete coverage of the cerebral vasculature [53]. Moreover, astrocytes regulate the local CNS blood flow by producing and releasing different molecular mediators, such prostaglandins (PGE), nitric oxide (NO), and arachidonic acid (AA) that can increase or decrease CNS blood vessel diameter [54]. Astrocytes have an active role in the metabolic support of neurons and respond to intensified neuronal activity by increasing the rate of glucose uptake, glycolysis, and the release of lactate into the extracellular space [55]. Astrocytes only need 10–15% of the total brain energy as compared to approximately 85% of the glucose required by neurons. These glial cells have higher rates of glycolysis but lower oxidative phosphorylation rates than neurons, suggesting a preference for the production of lactate [56,57]. In fact, glucose is absorbed by astrocytes that are in close contact with capillaries, and part of it is released by astrocytes as lactate into the extracellular space [58,59]. The lactate is the main substrate for neuronal functioning during cerebral activation, and through the astrocyte-neuron lactate shuttle (ANLS), is transported between astrocytes, which serve as a “lactate source”, and neurons which serve as a “lactate sink” [60,61]. In opposition to the ANLS hypothesis, Bak et al., [62] asserted that oxidative metabolism of lactate within neurons only occurs during repolarization rather than during neurotransmission activity and neurons use lactate as an “opportunistic” substrate when it is present. However, the emerging role of astrocytes has helped in settling this debate in favor of the ANLS hypothesis. The interactions among neurons and glia actively regulate brain homeostasis through proper synaptic plasticity and the release of neurotransmitters. Astrocytes, through astrocyte processes, establish close connections with neurons, in pre-and postsynaptic areas, forming the so-called “tripartite synapses”, a synapse composed of two neurons and an astrocyte that act synergistically as a functional unit. In a tripartite synapse, neurons release neurotransmitters that bind receptors expressed not only by neurons but also by adjacent astrocyte processes, whose activation leads to the increase of intracellular Ca^2+^ concentration, which in turn can spread to other neighboring astrocytes [63,64]. The increase of Ca^2+^ induces the release of neuroactive substances, called gliotransmitters, comprising glutamate, GABA, adenosine triphosphate (ATP), adenosine, D-serine, and BDNF. The gliotransmitters are secreted in a Ca^2+^ dependent manner, through vesicle and lysosome exocytosis to then activate neuronal receptors [65,66]. Therefore, whilst astrocytes cannot determine the propagating action potentials in the same way as neurons, they can communicate in a bidirectional manner with neurons and other astrocytes via the release of transmitters, exerting an active role in the storage and processing of information in the brain and modulating plasticity. Interestingly, the astrocytic 3-phosphoglycerate dehydrogenase (Phgdh)-dependent serine shuttle regulates the activation by d-serine and glycine of the N-methyl-D-aspartate receptors (NMDARs) which are involved in LTP [67]. Accordingly, Henneberger et al., [68], demonstrated that clamping internal Ca^2+^ in individual CA1 astrocytes blocks LTP induction in neighboring excitatory synapses, and such LTP blockade is reversed by exogenous D-serine or glycine supplementation. In physiological conditions, astrocytes are involved in providing protection to neurons. Through the tripartite synapses, astrocytes exert a key role in regulating the homeostasis of transmitters, including glutamate. In fact, these glial cells prevent neuronal excitotoxicity by controlling extracellular glutamate levels via excitatory amino acid transporters 1 and 2 (EAAT1, EAAT2, respectively) in humans, or glutamate/aspartate transporter (GLAST) and glutamate transporter-1 (GLT-1) in rodents [69,70]. Astrocytes can also remove excessive accumulation of the inhibitory neurotransmitters GABA through the GABA transporters (GATs), including GAT-1 and GAT-3, which are largely expressed in astrocyte processes [71]. The uptake of neuro-transmitters into astrocytes is followed by their conversion into the inactive precursors, which can then be recycled back to synapses where these are reconverted into active transmitters. This mechanism promotes the clearance of neurotransmitters from the synaptic space, ensuring a low level of extrasynaptic glutamate that prevents excitotoxicity and stimulates the release of anti-oxidants such as glutathione to protect neurons from oxidative stress [72,73]. Moreover, astrocytes express gap junctions through which cells are interconnected with each other [74]. These channels regulate the diffusion of nutrients and maintain the homeostasis of different ions, including K+. In fact, to avoid the toxicity associated with high extracellular concentrations of K+, astrocytes transfer high levels of K+ to adjacent astrocytes via gap junctions [75].

Astrocytes play an important role in the formation, maintenance, and pruning of synapses during development. They form molecular boundaries that guide the migration of developing axons, and through the release of molecular signals such as thrombospondin, promote the formation of new synapses [76,77]. Astrocytes also improve learning and memory, by increasing synaptic activity and strength [78]. Furthermore, astrocytes intervene in the regulation of the sleep−wake cycle and in the coordination of fine motor skills, and also modulate depressive-type behaviors and the effects of aging [79,80,81,82]. In summary, in the healthy CNS, astrocytes have multiple essential functions: they provide metabolic support to neurons via lactate production, maintain the homeostasis of fluid, ions, and transmitters, promote synaptic plasticity and functions, defend against oxidative stress, and secrete neuroprotective mediators. Astrocytes are actively engaged in all forms of CNS insults, such as during ischemia, trauma, infections, and neurodegenerative diseases (i.e., Alzheimer’s disease (AD) and Parkinson disease (PD)), through a process known as reactive astrogliosis, in which astrocytes are subjected to a continuum of progressive cellular and molecular changes whereby they lose their normal protective/homeostatic functions and acquire a phenotype that may lead to detrimental effects. On the other hand, astrocyte-based experiments represent a promising therapeutic strategy, applicable to different neurodegenerative disorders. In particular, the conversion of the midbrain astrocytes into new dopaminergic neurons, which provide axons to reconstruct the nigrostriatal circuit, can be a potentially powerful and feasible approach applicable to PD [83].

## 4. The Effects of Physical Exercise on Astrocytes

**Figure 3:**
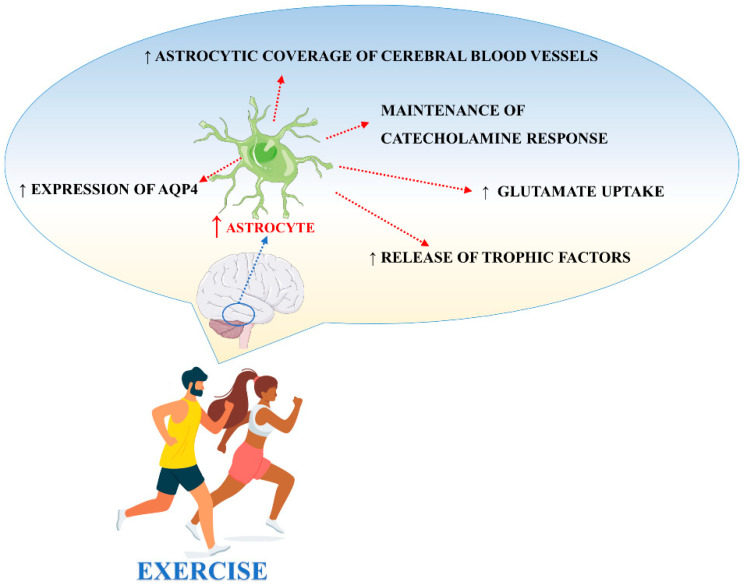
Main processes involved in exercise-induced modulation of astrocyte activities.

There are different studies in the literature focused on the effects of exercise on astrocytes. Exercise, through different biological mechanisms, promotes and protects neuronal function also via the adaptation of astrocyte activities (Figure 3). However, important aspects like animal species, age, and the brain structure analyzed, should be taken into consideration when interpreting the results [84].

It is well known that oxidative stress is a common feature of different neurodegenerative diseases, including AD, and astrocytes represent the main actors of the antioxidant response in the brain [85]. It has been shown that a five-week treadmill training of rats subjected to intracerebroventricular administration of streptozotocin (to model sporadic AD) increased the hippocampal content of astrocytic glutathione in CA1 region [86]. Furthermore, rats subjected to combined caloric restriction and moderate treadmill exercise consisting of 20 min, three times a week for twelve weeks, showed an increase in glutamate uptake from the extracellular space, suggesting a key role for astrocytes in avoiding excitotoxicity damage and eventually, neuronal death [87]. Belaya et al., investigated the positive role of exercise in 5xFAD mice, a well-known AD model featured by rapid and early onset Aβ plaque deposition (i.e., starting from 2 months of age). The results showed that voluntary physical exercise increased GFAP-positive astrocytes in the hippocampi compared with wild-type (WT) mice. In addition, astrocytes showed an enlarged soma area, solid shape, and presented with atrophic branches, and most of these cells were plaque-associated [88]. In contrast, long-term treadmill exercise reduced the number of hippocampal GFAP+ astrocytes in APP/PS1 mice model of AD, and voluntary running decreased the intensity and size of activated astrocytes in the cortex and hippocampus in the double transgenic APPswe/PS1ΔE9 mouse model of AD [89,90]. Moreover, ultra-endurance race simulation after high-volume training in rodents altered the GFAP isoform profile, suggesting impaired astrocyte reactivity in the cerebellum [91], where, in normal conditions, astrocytes regulate the homeostasis of this region, locomotor performance, and represent an antioxidant resource [92]. A combination of treadmill and running wheel for 4 weeks significantly increased the extent of astrocyte processes in adult mice. In addition to the increased extent of astrocytic processes following exercise, it appeared that these were preferentially directed towards the DG of the hippocampus [93]. The voluntary wheel running exercise performed by aged mice increased the expression of the astrocytic water channel aquaporin 4 (AQP4), a major component of the lymphatic–glymphatic system, which promotes paravascular cerebrospinal fluid (CSF)–interstitial fluid (ISF) exchange [94]. Interestingly, the AQP4 cell-surface abundance increases in response to hypoxia-induced cell swelling in a calmodulin-dependent manner [95], and the inhibition of AQP4 expression at gene and protein levels in poststroke animals reduces brain swelling and edema [96], suggesting the essential role of targeting astrocytes for the development of CNS edema therapies.

High levels of hippocampal GFAP, were found in diabetic animals following a running exercise using a treadmill apparatus, suggesting that physical training prevents and/or reverts diabetes-induced astrocytic GFAP reduction in the hippocampus [97]. Ferreira et al. (2011) observed that astrocytic plasticity after exercise, as demonstrated by the increase of GFAP in the hippocampus after 3 and 15 days of moderate exercise, was, in general, independent from transcriptional processes and BDNF upregulation. The exercise also induced the proliferation of astrocytes in the subgranular zone of the rat hippocampus [98,99] and the number of GFAP-expressing cells after 7 days of wheel running [100]. Voluntary wheel running also increased the number of new astrocytes in layer 1 of the motor cortex in healthy mice [101]. Moreover, exercise increased the number of GFAP-positive astrocytes in the frontoparietal cortex and striatum [102]. Saur et al., demonstrated that physical exercise increases the density of astrocytes and morphologically alters GFAP-positive astrocytes in the CA1 region of the hippocampus in healthy rats [103]. These data could be due to the capacity of exercise to increase FGF and NGF which are both able to induce astrocytic proliferation [104]. In contrast, it has been shown that moderate treadmill exercise (20 min/day) for 4 weeks decreased the GFAP content in the rat hippocampus [105]. Recently, the study demonstrated that 6 weeks of running exercise significantly increased the number of GFAP+ cells and the density of BrdU+/GFAP+ cells in the hippocampal CA1 region and DG in a rat model of depression, suggesting the role of exercise to promote the generation of new astrocytes [106]. Interestingly, high levels of cleaved caspase-3 positive astrocytes were found in hippocampal CA1 and DG regions after an acute bout of exercise [107]. In this context, cleaved caspase-3 did not exert its canonical role as executor in the apoptotic cell death process, but rather it seemed to regulate the neurogenesis and morphological changes of astrocytes in adult rats following exercise. Different studies have demonstrated that exercise induces cognitive improvements, especially on those tasks that involve the hippocampus and prefrontal cortex [108,109]. Brockett et al. demonstrated that rats subjected to a moderate duration of running (12 days) displayed enhanced performance on different cognitive tasks [110]. Furthermore, they found an increment in the size of the astrocyte cell bodies in response to running and this effect was specifically observed in the medial prefrontal cortex, orbitofrontal cortex, and hippocampus, suggesting the involvement of astrocytes in cognitive function. In agreement with these findings, the induction of an astrocyte-specific pathology in the medial prefrontal cortex by injection of an astrocyte-targeted toxin triggered neuronal damage that led to an impaired working memory, behavior flexibility, attention span, and reference memory in dedicated tasks [111]. Another study focused on the effect of exercise on Olig2-lineage astrocytes, specifically localized to certain regions of the gray matter including the globus pallidus. The results from these studies showed that voluntary exercise for 3 weeks induced astrocytic morphological changes, suggesting a response to motor activities [112]. Interestingly, Lundquist et al., [113] corroborated this theory by showing that exercise remodels astrocytes in an exercise- and region-dependent manner. In particular, following treadmill exercise, astrocytes within the prefrontal cortex increased GFAP expression, their distal-to-proximal span, and overall arborization following one week of exercise before returning to baseline morphology even with the continuation of exercise. This contrasts with results obtained in the striatum, showing that GFAP expression was initially decreased in astrocytes following one week of exercise to then increase and show changes in morphological complexity with the continuation of exercise. Other results have shown that early and long-term exercise, after cerebral hypo-perfusion induced by permanent bilateral occlusion of the common carotid arteries in rats, significantly increased the astrocytic coverage of cerebral blood vessels in the parietal cortex and CA1 region of the hippocampus [114]. Moreover, preconditioning exercise increased astrocyte proliferation and improved angiogenesis in the penumbra areas following brain ischemia [115], confirming the important role played by physical exercise on the neurovascular unit and highlighting how astrocyte coverage is essential for BBB integrity and for the cross-talk between neurons and vessels. Moreover, the upregulation of astrocytic end-feet contacts with blood vessels induced by exercise could be important to respond to intense energy demand, considering that these end-feet contain glucose transporters 1 (GLUT1) [116]. The effect of exercise on astrocytes is also mediated by catecholamines. Long-term physical activity promotes the maintenance of basal levels of noradrenaline, essential for inhibiting the release of proinflammatory cytokines by microglia [117,118] and for stimulating astrocytes to release trophic factors to afford neuroprotection [119].

## 5. Perspectives

The increase in life expectancy, a result of modern society, is accompanied by a higher risk of disease, disability, and especially cognitive decline. Therefore, it is essential to comprehend the mechanisms by which physical exercise may promote a healthy brain functionality. The evidence, above described, suggests that the effects of exercise on astroglia can play a pivotal role in providing such benefits. In fact, the astrocytes exert different roles to maintain an optimally suited milieu for neuronal functioning, by regulating redox potential, neurotransmitter release and ion concentrations, providing adequate metabolic support to neurons, regulating the production of trophic factors, and by providing defense mechanisms against oxidative stress. The development and the use of 3D cultures, cerebral organoids (COs), humanized self-organized models, microfluidic devices and human organ-on-a-chip platforms can represent valid instruments to support further discoveries related to astrocyte functions in physiological and pathological conditions [120,121,122]. Moreover, the use of efficient high-throughput screening and computer-aided drug design can give a novel insight supporting specific target validation for astrocyte-biomarkers (e.g., AQP4), since, to date no validated drugs are currently available for clinical use [123,124]. In conclusion, physical exercise is a valid and accessible tool to everyone to improve cognitive well-being via mechanisms that also involve astroglia. Although alone it cannot be used to treat neuro-pathologies, exercise represents an indisputable add-on therapy with great potential.

## Data Availability

Not applicable.
